# Elastic Compression Dressing after Total Hip Replacement Slightly Reduces Leg Swelling: A Randomized Controlled Trial

**DOI:** 10.3390/jcm13082207

**Published:** 2024-04-11

**Authors:** Sebastian Rohe, Sabrina Böhle, Georg Matziolis, Frank Layher, Steffen Brodt

**Affiliations:** Orthopedic Department of the Waldkliniken Eisenberg, Professorship of the University Hospital Jena, 07607 Eisenberg, Germany; s.boehle@waldkliniken-eisenberg.de (S.B.); g.matziolis@waldkliniken-eisenberg.de (G.M.); f.layher@waldkliniken-eisenberg.de (F.L.); s.brodt@waldkliniken-eisenberg.de (S.B.)

**Keywords:** total hip replacement, spica compression dressing, lymphedema, postoperative swelling, blood loss

## Abstract

**Background**: Even minor adverse reactions after total hip replacement (THR), including lymphedema, postoperative leg swelling, and blood loss, compromise patient comfort in times of minimally invasive fast-track surgery. Compression dressings are commonly used in surgical practice to reduce swelling or blood loss. However, the use of spica hip compression dressings after primary THR is controversial, and prospective studies are lacking. **Methods:** We conducted a prospective, single-center, two-arm, randomized controlled trial (RCT) of patients undergoing THR for primary osteoarthritis. A total of 324 patients were enrolled; 18 patients were excluded, and 306 patients were finally analyzed. Leg swelling as primary endpoint was measured pre- and postoperatively with a rotating 3D infrared body scanner. Secondary endpoints were transfusion rate and blood loss, estimated by Nadler and Gross formulas. **Results:** Postoperative leg swelling was lower in the compression group (241 ± 234 mL vs. 307 ± 287 mL; *p* = 0.01), even after adjustment for surgery time and Body-Mass-Index (BMI) (*p* = 0.04). Estimated blood loss was also lower in the compression group on the first (428 ± 188 mL vs. 462 ± 178 mL; *p* = 0.05) and third (556 ± 247 mL vs. 607 ± 251 mL; *p* = 0.04) postoperative days and leveled off on the fifth postoperative day, but lost significance after adjustment for BMI and surgery time. Neither group received a transfusion. **Conclusions:** Compression dressing after THR in the context of minimally invasive surgery slightly reduces leg swelling, but has no effect on blood loss or blood transfusion rate. So, this method could not generally be recommended in primary hip replacement.

## 1. Introduction

Total hip replacement (THR) is a common procedure, and major complications are rare. Minor adverse reactions include lymphedema, postoperative blood loss, and leg swelling. Pre-existing lymphedema and postoperative blood loss as a trigger for transfusion are known risk factors for perioperative complications, such as infections [1,2]. In a 2020 review, Aziz et al. showed a prevalence of major complications after elective THR of 2.8%. Minor complications occurred in 5.1% of cases. These included 0.14% deep vein thrombosis, 1.2% excessive seroma or hematoma and 0.3% superficial wound infection [3]. To reduce these complications, minimally invasive surgical techniques have been developed, and antifibrinolytic drugs are commonly used [4,5,6]. In their study, Johansson et al. showed a reduced average blood loss of 330 mL through the administration of tranexamic acid [6]. Liu et al. studied the blood loss after THR in 2011 and showed an intraoperative blood loss of approximately 500 mL and a total blood loss based on hemoglobin (Hb) calculation of approximately 1700 mL, implying a higher postoperative blood loss than intraoperative [7]. Compression is used in surgical practice to stop or prevent bleeding, and it is conceivable that external pressure on the THR wound postoperatively would reduce the blood loss. In trauma care, the use of a tourniquet to stop acute bleeding is common practice [8]. Lymphoedema is also a risk factor for postoperative complications, particularly infectious complications, as it disrupts venous return and lymph drainage and causes poorer localized soft tissue perfusion [9]. An improved outcome was also shown here through local compression therapy in other clinical disciplines. So, compression stockings are currently still one of the standard treatments for chronic lymphedema [10]. However, the use of compression dressings after primary total hip replacement to reduce these minor adverse reactions is controversial. Some previous articles have suggested that local wound compression with specific compression devices may reduce transfusion and wound drainage, but have not considered modern surgical strategies in detail [11,12,13]. The use of a compressive wrap dressing (spica dressing) had also been shown to reduce wound drainage [14]. Furthermore, Koval et al. demonstrated a reduction in the formation of tape blisters in the wound area after total hip replacement by using an elastic spica bandage [15]. Even in a recently published narrative review about wound dressings after hip and knee joint replacement in the Journal of Bone and Joint Surgery, no recommendation could be made regarding a spica dressing due to the lack of data [16]. Furthermore, patients with total knee arthroplasty (TKA) showed a reduction in pain in the first 8 h after surgery, as well as a better Range of Motion (ROM) and a lower Length of Stay (LOS). These results cannot be uncritically transferred to patients after THR [17].

Although the potential benefits of hip compression dressings include reduced swelling as well as lower transfusion rates and reduced wound drainage, there are potential disadvantages, such as time consumption, costs, and discomfort. Furthermore, there are currently no studies investigating a simple compression wrap dressing (spica dressing) in the context of modern minimally invasive surgical fast-track procedures and the administration of antifibrinolytics.

Therefore, we hypothesized that (1) the use of a simple wrap compression dressing (spica dressing) immediately after total hip replacement is associated with a reduction in postoperative leg swelling and (2) with a reduction in blood loss and transfusion rate.

## 2. Materials and Methods

**Figure 1:**
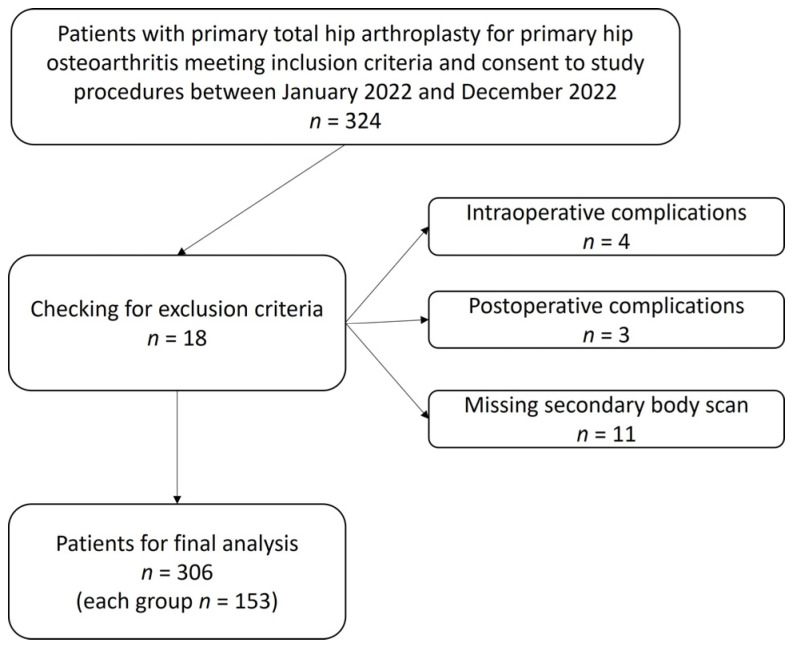
Flow chart of patient recruitment and exclusion.

A prospective, single-center, two-arm, randomized controlled trial (RCT) was conducted. A total of 324 patients undergoing total hip replacement for primary osteoarthritis were enrolled. The study was approved by the local ethics committee (2020-1963_1-BO) and registered in the German national registry of clinical studies (DRKS) with the number DRKS00022569. Informed consent was obtained from all patients. The specific patient flow from enrollment to analysis is outlined in Figure 1. Exclusion criteria were pregnancy, current malignant disease(s), secondary coxarthrosis due to dysplasia, post-traumatic and after previous hip surgery, chemotherapy 6 months prior to study entry, heart failure NYHA ≥ 4, history of drug abuse, congenital coagulation disorders, liver cirrhosis Child–Pugh class C; perioperative complications (intra- and postoperative, e.g., fracture, cup positioning problems, luxation and falls, as they could have an influence on blood loss and tissue reaction) and secondary diseases that do not allow participation in the in-house standard postoperative treatment regime after THR.

**Figure 2:**
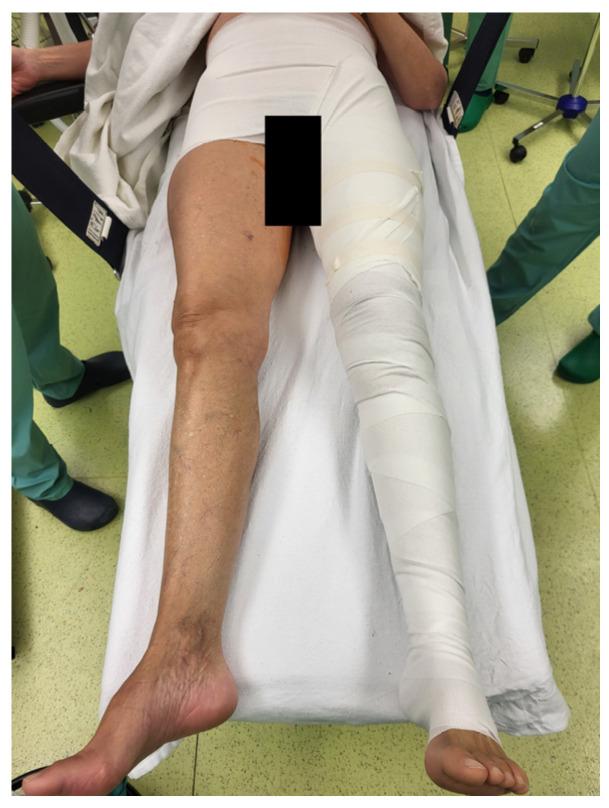
Compression dressing after total hip replacement in the compression dressing group (CDG).

All patients were operated on under general anesthesia in the supine or lateral decubitus position. A minimally invasive anterolateral or posterior approach was used according to surgeon preference [18,19,20]. Each patient received preoperatively 1 g of tranexamic acid intravenously and 1 g intraarticularly immediately before wound closure. The operations were performed by seven experienced hip surgeons. All of them were unselected staff members. All patients received cementless cups and stems. Cutaneous wound closure was performed with a stapler. The allocation order was generated using an Excel random number generator (Excel 2022; Microsoft, Redmond, WA, USA) according to simple randomization procedures. Patients were assigned to either the postoperative spica hip compression dressing group (CDG) or to the control group (CG) without spica hip compression dressing. An elastic spica hip compression dressing was applied to the entire operated leg, encircling the pelvis (Figure 2). The spica hip compression dressing was removed on the morning of the first postoperative day for early standardized mobilization protocol. As a result, a wearing time of 16 to 24 h was achieved, which corresponds to clinical practice. Components of perioperative care such as prophylactic antibiotics, thromboembolism prophylaxis (enoxaparin 4000 IE), pain management protocol, postoperative rehabilitation, and follow-up intervals were similar for all patients.

The primary endpoint was the difference in leg swelling depending on a hip compression dressing. Secondary endpoints were the estimated blood loss (EBL) on day 1, 3 and 5, blood transfusion rate and the C-reactive protein (CRP) course as a potential marker of tissue damage.

### 2.1. Leg Swelling

**Figure 3:**
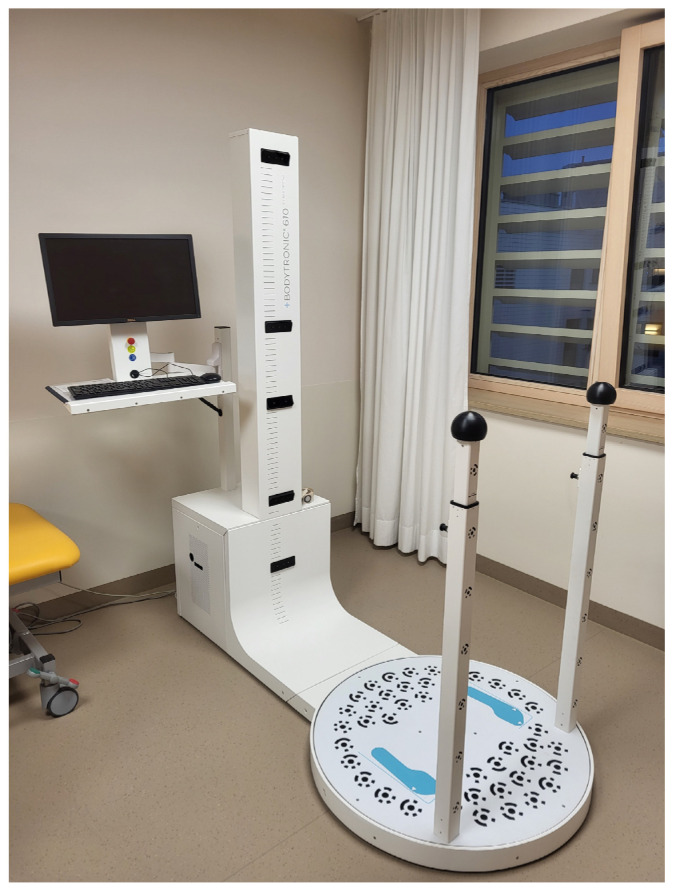
Bodytronic 610 infrared 3D scanner (Bauerfeind AG, Zeulenroda-Triebes, Germany).

Leg swelling represents the sum of lymphedema and perioperative bleeding into the thigh compartment. The volume (L) of the operated and unoperated leg was measured preoperatively and on the third postoperative day using a non-contact infrared scanning device, “Bodytronic 610” (Bauerfeind AG, Zeulenroda-Triebes, Germany) (Figure 3), validated by Tischer et al. with CT scans of a proof body [21]. The third postoperative day had to be chosen because it was necessary to stand on the rotation platform for 45 s without moving or shaking, and some patients were not yet able to do this on day 2. Subsequently, the pre- to postoperative differences of each leg were calculated. The variation of the unoperated leg was set as normal individual variation and subtracted from the difference of the operated leg.

### 2.2. Blood Loss

Hemoglobin was measured preoperatively and on postoperative days 1, 3, and 5. Transfusion of erythrocyte concentrates, wound healing disorders and anemic symptoms were documented in the hospital information system (HIS). Blood volume (BV) was estimated according to Nadler et al., taking into account sex, body mass and height [22,23]. Blood loss was calculated according to Gross’s formula: V_loss_ = BV ×(Hct_pre_ − Hct_po_)/Hct_ave_ where Hb_pre_ is the preoperative hematocrit, Hct_po_ is the postoperative hematocrit on postoperative days 1, 3, and 5, and Hct_ave_ is the average of Hct_pre_ and Hct_po_ [23].

Furthermore, postoperative C-reactive protein levels were recorded from the pre- and postoperative blood samples on day 1,3 and 5 to compare inflammation reaction in both groups and potential tissue damage [24,25,26]. If blood samples were also taken on day 2 and 4 additionally to the study protocol, they were also considered for calculating the course. The CRP value was measured in the hospital’s own laboratory using a qualitative visual latex agglutination test. A pathological CRP value was defined as higher than 5 mg/L (0.5 mg/dL). The effective measuring range of the method used is estimated to be 0.3 mg/L to 350 mg/L. The lowest limit of detection was 0.2 mg/L, and the lowest identification threshold was 0.3 mg/L. The effective measuring range and identification threshold were determined according to the explicit requirements of the medical device directive EP17-A of the Clinical and Laboratory Standards Institute [27].

### 2.3. Sample Size and Power

Power analysis was performed with GPower 3.1 (University of Kiel, Kiel, Germany) with an effect size of 0.4, an alpha error of 0.05 and a power of 0.95. Calculated group size was 136 patients and total sample size 272 patients. A total of 324 patients were finally included (Figure 1). Eighteen patients were excluded because of intraoperative complications (*n* = 4), postoperative complications (*n* = 3) and missing secondary body scans (*n* = 11), leaving 306 patients, 153 in each group, for final analysis.

### 2.4. Statistical Analysis

IBM SPSS Statistics 28 (IBM, Armonk, NY, USA) was used for statistical analysis. Data are presented as mean ± SD or range. Groups were compared using Student’s *t*-test or, when appropriate, Chi^2^- or Mann–Whitney U-test when data were not normally distributed. The normal distribution of metric data was examined using the Shapiro–Wilk test. Correlations between estimated blood loss, leg swelling, BMI and surgery time were tested with Pearson correlation coefficient and interpreted after recommendations from Mukaka et al. [28]. Covariations between confounders were assessed by ANCOVA analysis for surgery time and body mass index (BMI). Post hoc analysis of blood loss and leg swelling concerning BMI subgroups was performed, and confounders were considered. A *p*-level of 0.05 was considered significant; for multiple analysis in secondary endpoints, Bonferroni correction was performed.

## 3. Results

A total of 306 patients, 153 in each group, were finally analyzed. All patients tolerated the dressing until the next morning. However, some patients described discomfort due to the local pressure, while other patients felt stabilized by the dressing. But the individual experience has not been recorded systematically. There were no complications caused by the compression dressing like nerve palsy, skin lesions or surgical site infection. The control group also showed none of the complications mentioned. Medical history and anthropometric and surgical data of the patients are shown in Table 1, as well as the swelling of the operated leg, the estimated blood volume (EBV) and estimated blood loss (EBL) on the first, third and fifth postoperative day.

The control group (CG) and the compression dressing group (CDG) differed in terms of operation time and the following outcome parameters: leg swelling (primary endpoint) and EBL (secondary endpoint) on the first and third postoperative days (Table 1). Neither group received a transfusion.

Pearson correlation analysis showed a low to negligible correlation of EBL with BMI (day 1: r = 0.23, *p* < 0.01; day 2: r = 0.13, *p* = 0.02; day 3: r = 0.34, *p* < 0.01) and with surgery time (day 1: r = 0.23, *p* < 0.01; day 2: r = 0.31, *p* < 0.01; day 3: r = 0.26, *p* < 0.01), but no correlation between leg swelling and BMI (*p* = 0.49) or surgery time (*p* = 0.06). Therefore, BMI and surgery time were considered as confounders.

### 3.1. Leg Swelling

The increase in swelling of the operated leg as primary endpoint compared to the unoperated leg measured with an infrared rotating body scanner was lower in CDG (241 ± 234 mL vs. 307 ± 287 mL; *p* = 0.01) and remained significant after adjustment for BMI and surgery time (*p* = 0.04).

**Figure 4:**
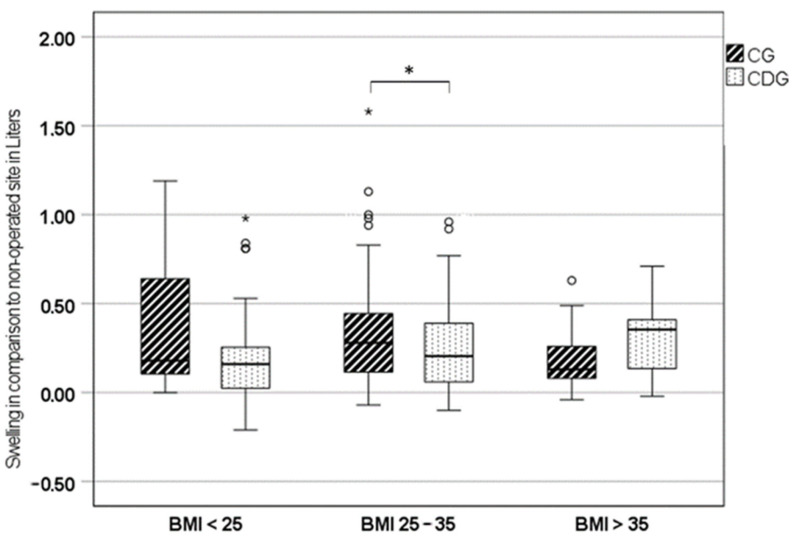
Box plots of the swelling of the operated leg in comparison to the non-operated leg of the control group (CG) and compression dressing group (CDG) with BMI sub-analysis after adjustment; * *p* ≤ 0.05.

After post hoc analysis for BMI subgroups, leg swelling was significantly lower in CDG for patients with BMI < 25 kg/m^2^ and between 25 and 35 kg/m^2^ (Table 2, Figure 4). After adjustment for confounders, leg swelling in patients with a BMI between 25 and 35 kg/m^2^ (*p* = 0.03) remained significant, while it lost significance in patients with a BMI < 25 kg/m^2^ (*p* = 0.21).

### 3.2. Blood Loss

**Figure 5:**
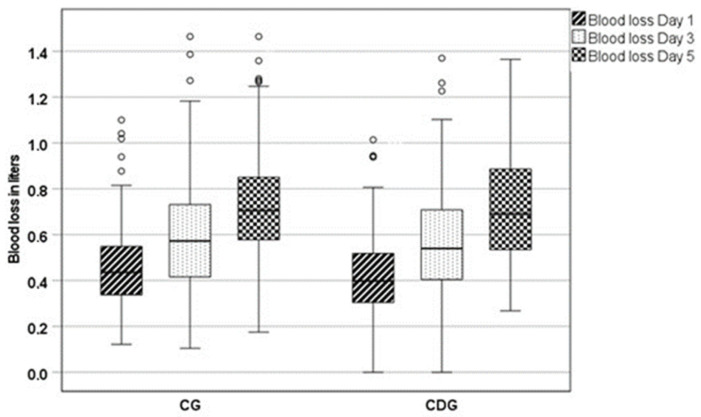
Box plots of the estimated blood loss (EBL) on postoperative days 1, 3 and 5, in liters [L], of the control group (CG) and compression dressing group (CDG) after adjustment for body mass index and surgery time.

The estimated blood volume (EBV) calculated by Nadler’s formula showed no difference in both groups. Estimated blood loss (EBL) calculated by Gross’s formula, which includes blood loss during surgery and hidden blood loss, showed a lower blood loss in the compression dressing group (CDG) on the first (428 ± 188 mL vs. 462 ± 178 mL; *p* = 0.05) and third (556 ± 247 mL vs. 607 ± 251 mL; *p* = 0.04) postoperative day compared to the control group (CG), losing significance after adjustment for BMI and surgery time and Bonferroni correction (day 1 *p* = 0.24 and day 3 *p* = 0.24) (Figure 5).

In the post hoc analysis of EBL concerning BMI subgroups, patients with BMI < 25 kg/m^2^ had a lower EBL on day 1, with a trend on day 3 and 5. Patients with BMI between 25 and 35 kg/m^2^ had also a trend toward a lower EBL in the CDG without reaching significance. With a BMI > 35 kg/m^2^, there was a trend toward higher blood loss in the CDG (Table 2). After adjustment and Bonferroni correction, EBL lost significance in patients with BMI < 25 kg/m^2^ on postoperative day 1 (*p* = 0.04).

There were no transfusions of erythrocyte concentrates in both groups.

### 3.3. C-Reactive Protein (CRP)

**Figure 6:**
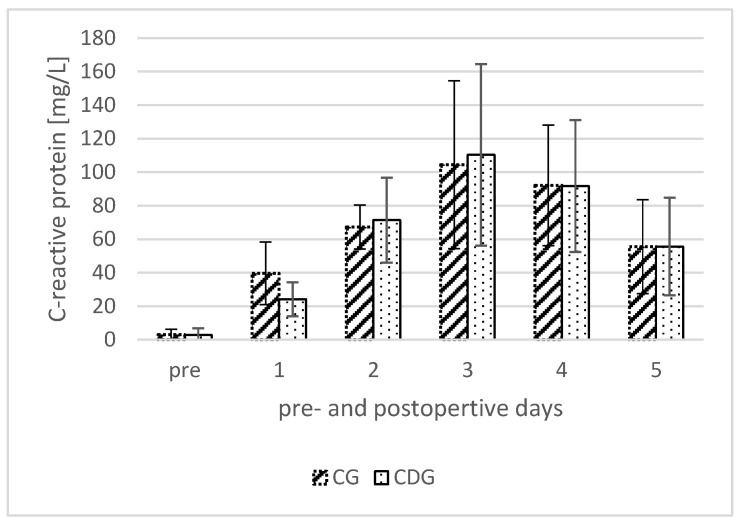
Course of pre- and postoperative C-reactive protein (CRP) levels in the control group (CG) and the compression dressing group (CDG).

Figure 6 shows the course of C-reactive protein (CRP) as secondary endpoint in both groups, with the well-known peak on the third postoperative day [29]. CRP levels, as a surrogate parameter for inflammatory response and surgical tissue damage, showed no significant difference in these groups (pre *p* = 0.72; postoperative day 1 to 5 *p* = 0.26; *p* = 0.86; *p* = 0.33; *p* = 0.96; *p* = 0.99, respectively) [24,25,26] (Figure 6).

## 4. Discussion

Our study is the first investigating postoperative leg swelling after total hip replacement using a 3D infrared scanner of the lower extremities within a modern fast-track surgery. We reported a slight reduction in leg swelling of approx. 65 mL even after adjustment, and no reduction in estimated blood loss or transfusion rate after adjustment. But looking at the amount of approx. 65 mL in comparison to the complete volume of one leg, the effect seems to be very low, so we think that an individual patient will not mention this reduction. On the other hand, in critically ill patients, even a small reduction in swelling and potential blood loss can help in reducing complications.

In a post hoc analysis with BMI subgroups, a reduction in leg swelling was seen only in patients with a BMI between 25 and 35 kg/m^2^, while there was no effect on EBL after adjustment. However, these post hoc results should be treated with caution due to the small number of cases in the subgroups and the nature of the post hoc analysis.

This study is the first to demonstrate a modest reduction in lower extremity swelling with compression dressing after total hip replacement. Previous studies investigating the reduction of swelling in the lower extremity after total hip replacement with compression dressing are lacking. For total knee arthroplasties (TKA), Matthews et al. showed no effect of elastic compression dressing, in contrast to our results, which could be explained by the different types of surgery and tissue damage [30]. Also, a meta-analysis resulted in no clear recommendations concerning compression bandages regarding the blood loss after TKA [31]. When considering the subgroups by BMI, a reduction in swelling of the operated leg was evident in those with a BMI between 25 and 35 kg/m^2^, with a volume difference of 75 mL. Patients with a BMI higher than 35 kg/m^2^ seemed to have greater swelling of the operated leg in the compression dressing group, without significance. This could be explained by a deterioration in lymphatic drainage and venous function of the lower limb in relation to BMI, explained by increased intra-abdominal pressure, which is already known and leads to lymphatic edema of the limb, which may gradually aggravate postoperative leg swelling and could explain our results especially in patients with high BMI [32,33,34]. In addition, compression up to the waist may increase intra-abdominal pressure and decrease lymphatic drainage and venous function in both legs.

Concerning the absolute estimated blood loss generally, this study showed a slightly lower estimated total blood loss than described by Cao et al., who reported a loss of 744 to 1102 mL depending on the dose of tranexamic acid used perioperatively through a posterolateral approach, and by Zha et al., who reported a blood loss of 737 to 1015 mL depending on the use of tranexamic acid through a direct anterior approach [35,36].

This study showed no difference in estimated blood loss between both groups after adjustment. In contrast, Tan et al. reported in 2016 a reduction in total and hidden blood loss and a lower transfusion rate (*n* = 6) in a small group of 17 patients with a compression dressing compared to 17 patients without compression [13]. Also, Johansson et al. reported in 2005 a lower transfusion rate by using an inflatable cuff for local compression but without administration of tranexamic acid, which can actually overshadow the effect of the compression dressing seen in our study by its well-investigated antifibrinolytic effect [6,11]. Looking at our subgroup analysis, the largest effect on EBL was seen in lean patients with a BMI less than 25 kg/m^2^ on the first postoperative day, and leveled off in the further course until the fifth postoperative day. Patients with a BMI greater than 25 kg/m^2^ showed no reduction in blood loss with the compression dressing, which may be explained by a more voluminous soft tissue sheath and thus insufficient pressure for deep hemostasis.

As our exclusion criteria were very restrictive and patients with current malignant disease(s), secondary coxarthrosis due to dysplasia, post-traumatic and after previous hip surgery, chemotherapy, heart failure NYHA ≥ 4, coagulation disorders, liver cirrhosis Child–Pugh class C, or perioperative complications were excluded, our results can only be applied to the general public to a limited extent. For example, patients with a coagulation disorder due to various pre-existing conditions could benefit from an additional compression dressing in the event of higher perioperative blood loss. Similarly, patients in whom a more extended approach has become necessary due to intraoperative complications could probably benefit from a compression bandage, too. Liu et al. also showed increased blood loss in patients with aseptic femoral head necrosis and dysplasia coxarthrosis [7]. Whiles these patients were excluded from our study population, it is also possible that they could benefit more from a compression dressing due to increased blood loss. Unfortunately, there is no current literature on this, and further studies are needed.

This study has several limitations. First, we were unable to blind the patients, operation room staff, and surgeons after wound closure. However, any study evaluating the use of a compression bandage after total hip replacement will be subject to these limitations. Second, blood loss was a calculated value using the Gross and Nadler formula based on hematocrit, which may be subject to calculation bias. Finally, the pressure of the compression dressing was not recorded. Therefore, we are uncertain whether the same effects of pressure were standardized across all study participants. However, it should be noted that standard practice is to apply compression bandages without recording pressure. Therefore, the same variation in pressure would be found in our study as in regular practice.

## 5. Conclusions

This study showed that a compression dressing after total hip replacement in the context of minimally invasive surgery only slightly reduces leg swelling (approx. 65 mL) but has no effect on initial postoperative blood loss or blood transfusion rate in a restricted patient population. In obese patients, it seems to have a negative effect on swelling and blood loss, without significance. So, this method could not generally be recommended in primary hip replacement. Further studies are needed to assess the benefits of compression dressings or alternative techniques to minimize leg lymphedema and blood loss in special patient groups, patients with certain pre-existing diseases, or critical ill patients, who were excluded here, so that they are not deprived of them. Also, while there is some evidence from knee arthroplasty, the effect on postoperative pain and early mobilization has not yet been conclusively clarified and needs further investigation.

## Figures and Tables

**Table 1:** Patients’ characteristics, economic, operation and outcome data.

	CDG	CG	*p*-Value	*p*-Value (Adjusted *)
Total	153	153	1.00	
Male sex	71 (46.4%)	65 (42.5%)	0.49	
Age [y]	64.07 ± 0.99	64.57 ± 11.75	0.81	
Obesity	38 (24.8%)	34 (22.2%)	0.59	
Overweight	118 (77.1%)	116 (75.8%)	0.87	
BMI [kg/m^2^]	28.66 ± 4.95	28.96 ± 5.34	0.31	
Diabetes	22 (14.4%)	21 (13.7%)	0.87	
Renal failure	6 (3.9%)	11 (7.2%)	0.21	
Osteoporosis	12 (7.8%)	11 (7.2%)	0.83	
Hypertension	97 (63.4%)	91 (59.5%)	0.48	
Coronary disease	8 (5.2%)	8 (5.2%)	1.00	
				
Length of stay [d]	5.93 ± 0.67	5.91 ± 0.84	0.38	
Surgery time [min]	51.39 ± 17.39	55.40 ± 21.76	**0.04**	
Anterolateral approach	130 (85.0%)	132 (86.3%)	0.87	
Dorsolateral approach	23 (15.0%)	21 (13.7%)	0.81	
Blood transfusion	0	0	-	
				
p.o. Leg swelling [L]	0.24 ± 0.23	0.31 ± 0.29	**0.01**	**0.04**
EBV [L]	5.01 ± 1.04	4.85 ± 0.97	0.09	-
EBL p.o. day 1 [L]	0.43 ± 0.19	0.46 ± 0.18	**0.05**	0.24
EBL p.o. day 3 [L]	0.56 ± 0.25	0.61 ± 0.25	**0.04**	0.24
EBL p.o. day 5 [L]	0.72 ± 0.26	0.73 ± 0.23	0.38	-

CDG = Compression dressing group; CG = Control group; BMI = body mass index; EBV = estimated blood volume; EBL = estimated blood loss; p.o. = postoperative; Overweight = BMI > 25 kg/m^2^; Obesity = BMI > 30 kg/m^2^; y = years; kg = kilogram; m = meter; d = days; min = minutes; L = liters; * adjusted for BMI and surgery time.

**Table 2:** Post hoc sub-analysis of estimated blood loss and leg swelling with BMI < 25 kg/m^2^, 25–35 kg/m^2^ and > 35 kg/m^2^.

	CDG	CG	*p*-Value
**BMI < 25 kg/m^2^ [*n*]**	35	36	
Leg swelling [L]	0.22 ± 0.28	0.35 ± 0.34	**0.04**
Blood loss p.o. day 1 [L]	0.34 ± 0.16	0.45 ± 0.17	**0.01**
Blood loss p.o. day 3 [L]	0.50 ± 0.24	0.61 ± 0.21	0.06
Blood loss p.o. day 5 [L]	0.62 ± 0.24	0.68 ± 0.17	0.14
**BMI 25–35 kg/m^2^ [*n*]**	96	106	
Leg swelling [L]	0.24 ± 0.22	0.32 ± 0.28	**0.02**
Blood loss p.o. day 1 [L]	0.45 ± 0.19	0.46 ± 0.18	0.33
Blood loss p.o. day 3 [L]	0.56 ± 0.23	0.60 ± 0.26	0.10
Blood loss p.o. day 5 [L]	0.72 ± 0.24	0.72 ± 0.23	0.47
**BMI > 35 kg/m^2^ [*n*]**	12	21	
Leg swelling [L]	0.31 ± 0.21	0.20 ± 0.18	0.06
Blood loss p.o. day 1 [L]	0.49 ± 0.20	0.49 ± 0.17	0.49
Blood loss p.o. day 3 [L]	0.68 ± 0.38	0.63 ± 0.31	0.33
Blood loss p.o. day 5 [L]	0.98 ± 0.27	0.82 ± 0.25	0.07

CDG = Compression dressing group; CG = Control group; BMI = body mass index; kg = kilogram; m = meter; L = liters; n = number; p.o. = postoperative.

## Data Availability

Individual participant data that underlie the results reported in this article, after deidentification, are available for researchers, who should direct requests to the corresponding author. To gain access, data requestors will need to sign a data access agreement. Data are available for 5 years.
